# Structural and Functional Analysis of Hemoglobin Binding to the Peritrophic Matrix During Blood Digestion in *Aedes aegypti*

**DOI:** 10.3390/insects16020116

**Published:** 2025-01-24

**Authors:** Karla Barreto da Silva Orozimbo, Desiely da Silva Gusmão Tauil, Aline Melila Licurgo, Felipe Figueirôa Moreira, Jucélia da Silva Araújo, Maria Aparecida Aride Bertonceli, Sérgio Henrique Seabra, Olga Lima Tavares Machado, Francisco José Alves Lemos

**Affiliations:** 1 Laboratório de Biotecnologia, Centro de Biociências e Biotecnologia, Universidade Estadual do Norte Fluminense Darcy Ribeiro, Campos dos Goytacazes 28013-602, RJ, Brazil; 2 Instituto Federal de Educação, Ciência e Tecnologia Fluminense, Campos dos Goytacazes 28030-130, RJ, Brazil; desielygusmao@yahoo.com.br; 3 Laboratório de Química e Função de Proteínas e Peptídeos, Centro de Biociências e Biotecnologia, Universidade Estadual do Norte Fluminense Darcy Ribeiro, Campos dos Goytacazes 28013-602, RJ, Brazil; alinemelila@gmail.com (A.M.L.); jucelia@uenf.br (J.d.S.A.); cidaride@hotmail.com (M.A.A.B.); olga@uenf.br (O.L.T.M.); 4 Laboratório de Biologia Celular e Tecidual, Centro de Biociências e Biotecnologia, Universidade Estadual do Norte Fluminense Darcy Ribeiro, Campos dos Goytacazes 28013-602, RJ, Brazil; felipefmoreira91@gmail.com (F.F.M.); seabrash@uenf.br (S.H.S.)

**Keywords:** *Aedes aegypti*, blood digestion, hemoglobin, heme, chitin

## Abstract

The *Aedes aegypti* mosquito is responsible for transmitting pathogens such as the Dengue, Zika, and Chikungunya viruses. The peritrophic matrix (PM) is an extracellular chitin-rich structure that lines the midgut of arthropods, providing a crucial protective barrier for the gut epithelium against mechanical damage, ingested pathogens, and toxic substances. During blood digestion, hemoglobin is lysed, releasing free heme into the midgut lumen. Part of this heme binds strongly to the PM, mitigating its harmful effects on the mosquito epithelial cells. Our study focused on investigating the interaction dynamic between hemoglobin and the PM during blood digestion in *A. aegypti*. Optical microscopy was employed to observe the temporal progression of blood digestion in the *A. aegypti* midgut, highlighting significant morphological changes in the blood bolus. An electrophoresis analysis revealed distinct protein bands in the PM extract, some of which were associated with hemoglobin and its subunits. The presence of PM-associated hemoglobin was confirmed by amino-terminal sequencing and an immunoblot analysis using anti-hemoglobin antibodies. Furthermore, fluorescence microscopy revealed overlapping labeling between hemoglobin and chitin, suggesting an interaction between hemoglobin and PM chitin. Corroborating these results, hemoglobin showed an affinity with chitin in the chromatography and molecular docking assays, in which the hemoglobin subunits interacted with the oligosaccharide (NAG)_4_. Thus, hemoglobin may perform a function similar to that of peritrophins. Further experiments demonstrated the protective role of the PM against hemoglobin proteolysis during blood digestion. Overall, this study provides valuable insights into the intricate interactions between hemoglobin and the PM, enhancing our understanding of mosquito digestive physiology and potentially contributing to the development of vector control strategies.

## Simple Summary

The mosquito *Aedes aegypti* spreads diseases like Dengue, Zika, and Chikungunya, posing a significant threat to public health. Controlling this mosquito is essential for reducing disease transmission. Inside its midgut, a protective structure called the peritrophic matrix helps shield the gut lining during blood digestion. When mosquitoes consume blood, hemoglobin is broken down, releasing a molecule called free heme that can damage the gut lining and cause oxidative stress. Our study examined how hemoglobin interacts with the peritrophic matrix to minimize these harmful effects. We found that hemoglobin binds to chitin, a key component of the peritrophic matrix, in a way similar to structural peritrophic matrix proteins. This interaction reduces the breakdown of hemoglobin, thereby limiting the release of free heme and lowering oxidative stress in the mosquito’s gut. By understanding these processes, we gain valuable insights into mosquito biology that could inform innovative strategies to control *A. aegypti* populations. Such strategies may help reduce the spread of dangerous diseases, benefiting global public health.

## 1. Introduction

Mosquitoes are vectors of several infectious diseases that significantly impact human and animal health. These diseases, including dengue, yellow fever, malaria, and filariasis, are particularly prevalent in regions such as Latin America and Africa [1]. In 2023, global reports of dengue reached a record high, with over five million cases and more than 5000 deaths [2]. Furthermore, *A. aegypti* transmits key pathogens, such as the Zika and Chikungunya viruses, which are major contributors to morbidity and mortality worldwide [3], underscoring its critical importance in public health.

The peritrophic matrix (PM) is a chitin-based extracellular structure in the midgut of arthropods, acting as a semi-permeable barrier [4,5]. In blood-feeding insects like mosquitoes, it forms a protective sleeve around the blood bolus, separating it from the midgut epithelium [6]. The PM exists in two types: Type I, produced in response to feeding, and Type II, continuously secreted by specialized cells [7].

The PM provides physical protection for epithelial cells by preventing mechanical damage, acting as a selective barrier to pathogens, toxins, and other harmful substances [8]. It also defends against pathogens by limiting their access to the midgut and maintaining gut homeostasis [9,10]. In addition to its protective functions, the PM optimizes digestion by facilitating the compartmentalization of digestive processes and enhancing enzymatic efficiency [11]. The peritrophic matrix significantly influences vector competence through multiple mechanisms. The heme-peroxidase HPx1 plays a crucial role by promoting PM assembly and modulating arbovirus vector competence in mosquitoes [12]. Beyond viral pathogens, the PM serves as a critical barrier against *Plasmodium* parasites, directly affecting their development and transmission potential [13]. This protective function is enhanced by the gut microbiota, which stimulates PM formation through immune signaling pathways, thereby maintaining midgut homeostasis and preventing systemic infections [12,14]. The complex interplay between the PM, gut microbiota, and immune responses represents a sophisticated defense mechanism that modulates vector competence for various pathogens [13,14].

The PM comprises proteins, chitin, and other polysaccharides, with proteins being a major structural and functional component [15]. Peters [16] emphasized the dominance of proteins in the PM, while Tellam [17] introduced a classification system categorizing PM proteins based on their association with the structure. Among these, class III proteins, known as peritrophins, are particularly abundant. Peritrophins bind strongly to the PM through chitin-binding domains (CBDs), which contain cysteine residues forming disulfide bonds that stabilize the matrix [18].

Vertebrate hemoglobin (Hb) is a sophisticated tetrameric protein that serves as the primary oxygen carrier within erythrocytes [19]. The molecule consists of four globin chains arranged in a quaternary structure: two alpha (α) subunits with 141 amino acid residues (approximately 15.2 kDa each) and two beta (β) subunits with 146 amino acid residues (approximately 15.8 kDa each) (Mozzarelli et al., 2010), resulting in a total molecular mass of about 64 kDa for the complete tetramer. Each subunit contains a prosthetic heme group—an iron-containing protoporphyrin IX that enables reversible oxygen binding [20,21].

During blood digestion in hematophagous insects, erythrocyte lysis releases significant amounts of free heme into the midgut lumen [22,23]. While heme is essential for various biological functions, such as oxygen transport and signal transduction [24], free heme is toxic due to its ability to catalyze the oxidation of biomolecules, including nucleic acids, proteins, and lipids [25,26].

Studies have shown that the PM plays a crucial role in mitigating heme toxicity. Páscoa et al. [23] demonstrated that a portion of the heme released during blood digestion binds strongly to the PM, preventing it from reaching the gut epithelium and causing oxidative damage. Devenport and colleagues [22] identified AeIMUC1 as a key peritrophic matrix protein in *A. aegypti* that possesses dual functionality.

This protein contains three chitin-binding domains (CBDs) with an intervening mucin-like domain and can bind significant amounts of heme in vitro, suggesting its crucial role in heme detoxification during blood digestion.

Blood digestion in *A. aegypti* involves several coordinated processes, including water removal, erythrocyte disruption, macromolecule hydrolysis, and the absorption of nutrients by midgut epithelial cells [27]. Digestion occurs in two phases: an early phase (4–6 h post-feeding) with low trypsin activity facilitating limited proteolysis and a late phase (8–36 h post-feeding) characterized by a high activity of trypsin [28].

During the initial hours of digestion, when protease activity is low, the PM may bind intact or partially degraded hemoglobin. As digestion progresses, the mature PM predominantly binds free heme, reducing its diffusion to epithelial cells. This dual capacity highlights the PM’s role in sequestering hemoglobin-associated and free heme, mitigating oxidative damage during blood digestion.

This study investigated the interaction between hemoglobin and the peritrophic matrix, offering a deeper understanding of the digestive physiology of *A. aegypti*.

## 2. Material and Methods

### 2.1. Mosquitoes and Colony Maintenance

The insects were sourced from a colony of *A. aegypti* (Rockefeller strain) maintained at the Laboratory of Biotechnology, Universidade Estadual do Norte Fluminense Darcy Ribeiro-UENF, Campos dos Goytacazes, RJ, Brazil. The larvae were fed with Tetramin fish food and reared in trays at room temperature. The adult mosquitoes were housed at 27 °C with a relative humidity of 70%, under a 12 h photoperiod, and provided with a 10% sucrose solution. To stimulate egg production, females aged 3–5 days were artificially fed with defibrinated sheep blood using a membrane-feeding apparatus (CEUA/CBB/UENF approval no. 011/2023).

### 2.2. Feeding of Mosquitoes

For experiments involving blood feeding, the mosquitoes were fed using gently immobilized Balb/c mice, following approved ethical protocols (CEUA/CBB/UENF approval no. 011/2023), which adhere to the guidelines established by the National Council for Control of Animal Experimentation (CONCEA).

For artificial feeding, sheep blood plasma was supplemented with various proteins and offered to the mosquitoes using a water-jacketed membrane-feeding apparatus at 37 °C. The proteins used in the experiments were (a) bovine hemoglobin (H2500, Sigma-Aldrich, City of Saint Louis, MO, USA), (b) bovine serum albumin (B14, Thermo Scientific, Waltham, MA, USA), (c) bovine trypsin (Sigma-Aldrich, T8003), (d) soybean trypsin inhibitor (101113, MP Biomedicals, Singapore) 10 mg/mL, (e) bovine cytochrome C (C3131, Sigma-Aldrich, City of Saint Louis, MO, USA) 70 mg/mL, and (f) equine myoglobin (M1882, Sigma-Aldrich, City of Saint Louis, MO, USA).

### 2.3. Dissection of A. aegypti Midgut and Processing of the Peritrophic Matrix

The mosquitoes were blood-fed, and at specific time points post-blood feeding, the mosquitoes were immobilized in a freezer for 3 min at −20 °C and transferred to Petri dishes on ice. Dissections were performed in either 50% ethanol or PBS 1X under a Karl Zeiss stereoscopic microscope (Karl Zeiss, Oberkochen, Germany). The midgut was carefully exposed by gently pulling the last abdominal segment while holding the thorax with fine forceps. The isolated midgut was transferred to fresh buffer, where a precise incision was made to expose the blood bolus. The epithelial layer was carefully removed using fine forceps while preserving the integrity of the peritrophic matrix structure. The isolated peritrophic matrices were thoroughly washed with distilled water to remove residual blood content through a combination of mechanical disruption with a pestle and vortexing. The samples were centrifuged at 13,000× *g* for 5 min, and this washing procedure was repeated five times until the supernatant became colorless. Clean peritrophic matrices were either processed immediately or stored at −70 °C for later use. For the protein analysis, the samples were homogenized in appropriate buffer and separated by denaturing electrophoresis. For the morphological analysis, the midguts with blood bolus were photographed to document the structural changes in the midgut throughout the digestive process.

### 2.4. Immunofluorescence Microscopy

The midguts from the mosquitoes fed on sheep’s blood or bovine hemoglobin were fixed in 4% paraformaldehyde. They were then washed three times for 30 min each in PBS and were transferred, embedded in Optimal Cutting Temperature (OCT), and frozen in liquid nitrogen. Thereafter, cryosections (40 μm thick) were obtained with a Hyrax C25 cryostat (Zeiss, Stuttgart, Germany) at −20 °C and placed onto gelatin 0.5%-treated glass slides. The slides were permeabilized in 50 mM ammonium chloride, blocked with 3% bovine serum albumin (BSA), and subsequently incubated for 24 h at 15 °C with an anti-human hemoglobin primary antibody (H4890; dilution 1:100, Sigma-Aldrich, City of Saint Louis, MO, USA) in PBS containing 3% BSA. The samples were then washed three times with PBS (5 min each), blocked with 3% BSA, and incubated with the secondary antibody (Sigma-Aldrich, A3687; 1:500 dilution) in PBS for 24 h at 15 °C. This was followed by three additional PBS washes. Finally, the slides were stained with Calcofluor White (Sigma-Aldrich, 18909), a fluorescent blue dye that binds to chitin, and analyzed and photographed using an Olympus BX60 epifluorescence microscope (Tokyo, Japan). The samples prepared with sheep’s blood were examined using a Zeiss LSM-710 confocal microscope, and the images were processed and assembled using Zeiss Zen Lite 2.1 software.

### 2.5. Affinity Chromatography of Bovine Hemoglobin and Hemin with Chitin

To evaluate the potential interactions between bovine hemoglobin and chitin, as well as between the hemin (Sigma-Aldrich, H-9039) and chitin (Sigma-Aldrich, C-9213), affinity chromatography was performed using chitin as the matrix. Initially, 1 mg of bovine hemoglobin or bovine hemin was dissolved separately in 1 mL of sodium acetate buffer (0.1 M, pH 6.0) and incubated with 8 mL of hydrated chitin resin under constant agitation at room temperature for 30 min. The mixture was then loaded onto a glass chromatography column (2 × 15 cm), and the column was packed using a pressure pump with a constant flow rate of 1.0 mL/min. The non-retained fraction was eluted with sodium acetate buffer (0.1 M, pH 6.0) until the absorbance at 280 nm reached near-zero values, indicating the removal of unbound proteins from the chitin. The proteins retained on the column were eluted with 0.1 M HCl, chosen for its ability to disrupt protein–chitin interactions. Eluates were collected in 1 mL fractions and monitored for protein content by measuring absorbance at 280 nm using a Belphotonics spectrophotometer (BEL Engineering, Monza, Italy).

### 2.6. Computational Approach for Structural Modeling and Blind Docking of Bovine Hemoglobin Subunits with the Oligosaccharide (NAG)_4_

The FASTA sequence of bovine hemoglobin (PDB ID: 2QSS) was used to validate and obtain the three-dimensional models of the alpha and beta subunits in PDB format. The structural modeling of each subunit was performed using SWISS-MODEL (available at https://swissmodel.expasy.org/interactive “URL (accessed on 27 October 2024)” employing the crystal structure of bovine hemoglobin (PDB ID: 2QSS) as a structural model. The molecular docking of the alpha and beta subunits with the oligosaccharide (NAG)_4_ was conducted using the DockThor v.2 software. A blind docking approach was employed, defining the search space as a cube with dimensions 40 Å × 40 Å × 40 Å, sufficient to encompass potential binding sites across the protein surface. The three-dimensional model of the ligand (NAG)_4_ was constructed in PyMOL using the Azahar plug-in, specifically for oligosaccharide projection. During the procedure, all the ligand rotations were set to free, while the protein atoms remained rigid. The standard DockThor algorithm was used with parameters adjusted for 1,000,000 evaluations and 24 runs per experiment. The most stable protein–ligand complex was selected based on the affinity energy. Protein–ligand interactions were analyzed in PyMOL, focusing on hydrogen bonds, interaction distances (measured in Å), and amino acids at the binding interface, specifically LYS, ARG, and HIS, which are critical for stabilizing the complex.

### 2.7. Extraction and Quantification of Peritrophic Matrix Proteins from A. aegypti

The solutions used in the progressive protein extractions from PM were distilled water; Tris-HCl buffer 100 mM pH 7.5 containing NaCl 150 mM, EDTA 5 mM, and PMSF 0.1 mM; Tris-HCl buffer 20 mM pH 7.4 containing NaCl 140 mM, Triton X-100 1.5%, and PMSF 0.1 mM; Tris-HCl buffer 20 mM pH 7.4 containing NaCl 140 mM, urea 6 M, PMSF 0.1 mM; and 0.125 M Tris-HCl sample buffer pH 6.8 containing 20% glycerol, 10% SDS, 10% 2-β-mercaptoethanol, and 0.004% bromophenol blue. The volume of buffer added to the PMs for each extraction was 1.0 mL, regardless of the number of PMs used. Following each extraction, the samples were homogenized using a pestle and centrifuged at 13,000× *g* for 5 min at 4 °C in a refrigerated centrifuge. The samples were dialyzed in distilled water, in a volume of 1 L, for eighteen hours, with exchanges at intervals of 6 h. Finally, the samples were lyophilized.

After dialyzing and lyophilizing each sample obtained from the described extractions, the protein content was measured using the Folin–Ciocalteu method, as outlined by Lowry et al. [29]. The total protein concentration was estimated in the crude extract, the different extraction fractions, and the final precipitate.

Trichloroacetic acid (TCA) was added to the PM protein samples to a final concentration of 10%, promoting protein precipitation by acid-induced denaturation. The samples were then incubated on ice for 1 h to enhance the precipitation efficiency. Then, centrifugation was performed at 1650× *g* for 20 min at 4 °C in a 15 mL centrifuge tube, and the supernatants were discarded. The precipitated samples were thoroughly washed with 1 mL of pure ethanol to ensure the complete removal of residual TCA. Following the ethanol wash, the samples were transferred to Eppendorf-type tubes, where they were carefully homogenized and subjected to centrifugation at 22,000× *g* for 5 min at 4 °C to isolate the purified precipitate. The supernatants were discarded. After these steps, 1 mL of pure acetone was added to the precipitates. The samples were homogenized, centrifuged at 22,000× *g* for 5 min at 4 °C in a refrigerated centrifuge, and the supernatants were discarded. The tubes containing the final precipitates were left open for acetone evaporation. Finally, 20 μL of sample buffer was added to the final precipitates, followed by boiling and rapid centrifugation.

### 2.8. Electrophoresis, Detection of Peroxidase Activity, and Protein Quantification by Densitometry

TCA-precipitated proteins were separated using SDS-PAGE 15% [30], SDS-Tricine 10%T, 3%C [31]. After electrophoresis, the gels were stained with Coomassie Blue R (0.1% Coomassie Blue R, 40% methanol, and 10% acetic acid), silver nitrate, or dimethoxybenzidine (DMB), depending on the objective. Coomassie Blue R and silver nitrate staining were used to visualize the total protein content, followed by destaining with 40% methanol and 10% acetic acid to enhance the contrast. DMB staining was applied specifically for the detection of peroxidase activity.

The detection of peroxidase activity was performed as described by Francis and Becker [32]. After electrophoresis, the gels were incubated with 12.5% trichloroacetic acid (TCA) for 30 min, washed with distilled water for another 30 min, and developed in 100 mL of 0.05 M sodium citrate buffer (pH 4.4) containing 0.1 g of DMB and 200 μL of H_2_O_2_. The samples analyzed included peritrophic matrices (PMs) extracted from *A. aegypti* mosquitoes fed on blood for 48 h or subjected to diets containing plasma + albumin, plasma + hemin, and plasma + hemoglobin. Bovine hemoglobin was used as a control.

The densitometric analysis was performed using ImageJ 1.54m software (NIH, Bethesda, MD, USA) to quantify the protein band intensities in the SDS-PAGE gels. The 15% polyacrylamide gels were digitally scanned and converted to 8-bit grayscale images. For each gel, the background was calibrated and subtracted using a rolling ball radius of 50 pixels. The band intensities were measured using rectangular selections of equal size for the 14 kDa bands across all lanes. The integrated density function was used to obtain intensity values, with local background subtraction applied for each measurement. The values were normalized using the highest intensity band as 100% reference, and all other measurements were calculated as relative percentages. To ensure accuracy, the measurements were performed in triplicate, and only bands within the linear range of detection were included in the analysis. The resulting values were plotted as bar graphs showing relative band intensities (%) for each experimental condition. This standardized methodology allowed for the reliable quantification and comparison of protein levels across different samples and treatments.

### 2.9. Amino-Terminal Sequencing of the Majority PM Proteins

After electrophoresis, the proteins from adult PM were transferred to a PVDF membrane using a transfer buffer (50 mM Tris-HCl, 50 mM boric acid) at 28 V for 16 h. Prior to the transfer, the membrane was activated in 100% methanol, rinsed with ultrapure water, and equilibrated in transfer buffer alongside the gel for 20 min.

Following the transfer, the membrane was stained with Coomassie solution (40% methanol, 10% acetic acid, and 0.1% Coomassie R-250). The corresponding protein bands were excised and subjected to sequencing by automated Edman degradation, performed using a Shimadzu liquid-phase sequencer. The resulting phenylthiohydantoin derivatives of amino acids were identified via a Shimadzu HPLC system equipped with a Wakopak WS-PTH reverse-phase analytical column.

## 3. Results

### 3.1. Dynamic Visualization of Midgut Morphology During Blood Digestion in A. aegypti

**Figure 1:**
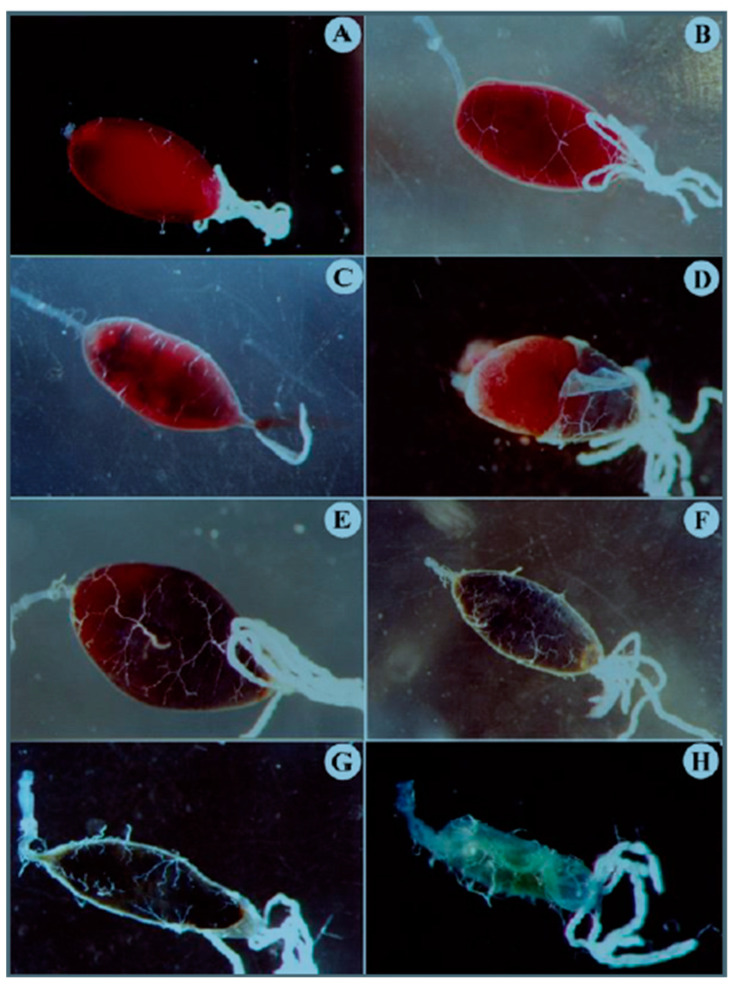
Temporal analysis of *A. aegypti* midgut morphology during blood digestion by optical microscopy. Images show the progressive changes in the blood bolus at different time points after blood feeding: (**A**) 30 min, showing initial blood meal encapsulation; (**B**) 4 h and (**C**) 6 h, demonstrating complete blood bolus formation; (**D**) 16 h, with partially removed intestinal epithelium revealing the PM-enclosed blood bolus; (**E**) 22 h and (**F**) 30 h, showing progressive darkening and compaction of the blood bolus; and (**G**) 54 h and (**H**) 72 h, displaying advanced digestion stages with significant reduction in bolus size. Note the characteristic network-like structure of the PM visible throughout the digestion process. Magnifications: (**A**–**D**,**F**): 25×; (**E**,**G**): 40×; and (**H**): 66×.

We used optical microscopy to observe the digestion of blood in the midgut of mosquitoes at different times after feeding (Figure 1). Right after the mosquitoes fed (Figure 1A), before the peritrophic matrix (PM) had formed, the blood was in direct contact with the stretched intestinal lining, making it difficult to distinguish the epithelium. By 4 h after feeding (Figure 1B), the epithelium became clearly distinguishable from the blood. Between 6 and 22 h after feeding, the midgut gradually darkened, a process associated with the breakdown of hemoglobin and the release of free heme (Figure 1C–F). In Figure 1D, when part of the intestinal epithelium was removed, we observed that the PM contained the food bolus. By 54 h (Figure 1G), the blood was fully digested, and the midgut appeared uniformly dark due to the presence of oxidized heme.

### 3.2. Protein Profiling of the Peritrophic Matrix in Blood-Fed Mosquitoes

**Figure 2:**
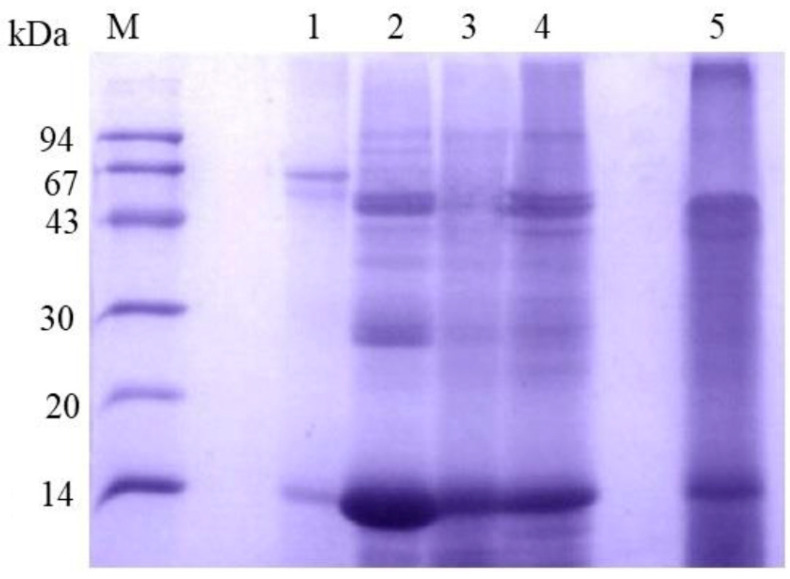
SDS-PAGE analysis of sequential protein extraction from *A. aegypti* peritrophic matrix (PM) using solutions with varying extraction efficiencies. Lane M: molecular mass markers (94, 67, 43, 30, 20, and 14 kDa). Extraction conditions: lane 1: water extraction; lane 2: Tris-HCl/NaCl buffer containing EDTA and PMSF; lane 3: Tris-HCl/NaCl buffer containing Triton X-100 and PMSF; lane 4: Tris-HCl/NaCl buffer containing urea and PMSF; and lane 5: Laemmli sample buffer. Note the differential protein extraction patterns, particularly for proteins between 14–94 kDa, demonstrating varying extraction efficiencies of different buffer compositions. The 14 kDa band is notably present across different extraction methods, suggesting its consistent association with the PM.

**Figure 3:**
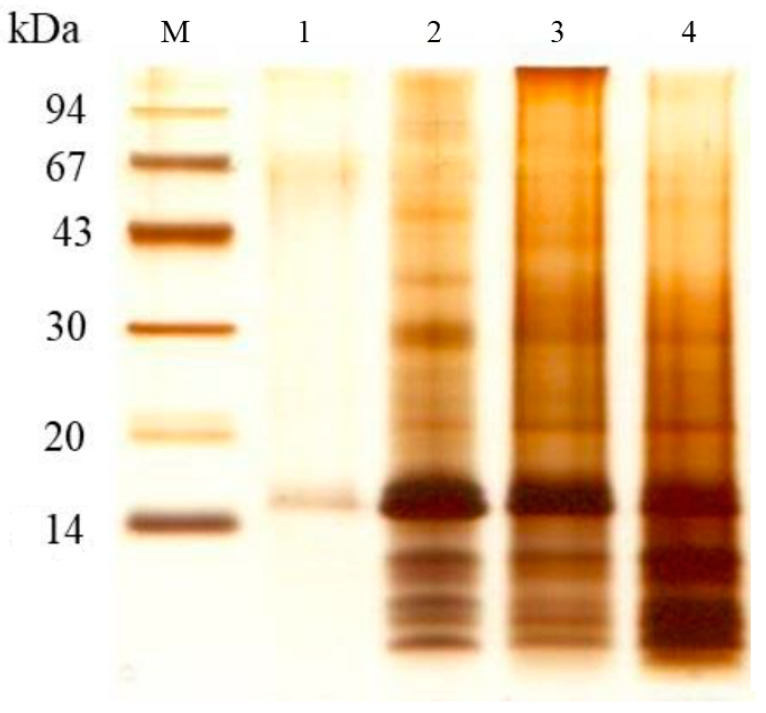
Tricine-SDS-PAGE analysis of sequential protein extraction from the peritrophic matrix of *A. aegypti* using solutions with varying extraction efficiencies. The gel contained a total concentration of 10% acrylamide and bis-acrylamide (10%T) and a crosslinker ratio of 3% (3%C). The proteins were visualized using silver nitrate staining. Lane M: molecular mass markers (94, 67, 43, 30, 20, and 14.4 kDa). Extraction conditions: lane 1: water extraction; lane 2: Tris-HCl/NaCl buffer containing EDTA and PMSF, showing enhanced protein extraction; lane 3: Tris-HCl/NaCl buffer containing urea and PMSF, demonstrating differential protein solubilization; and lane 4: Laemmli sample buffer extraction. Note the distinct protein profiles obtained with each extraction method, particularly in the low-molecular-weight range, revealing the complexity of PM-associated proteins and the efficiency of different extraction protocols.

To better understand the protein composition of the PM in blood-fed mosquitoes, we analyzed it using denaturing electrophoresis. After thoroughly washing the PMs with distilled water, we performed sequential extractions with buffers of increasing solubilization power (Figure 2). We observed several distinct protein bands, with the most prominent appearing at approximately 43, 30, and 14 kDa. These bands consistently appeared in different extraction steps, suggesting that the associated proteins interact with the PM through various mechanisms. To obtain a clearer view of the low molecular weight proteins, we used tricine gel electrophoresis (Figure 3). The tricine gel electrophoresis samples were the same as those used in the standard Laemmli gel electrophoresis shown in Figure 2, with the exception of the sample extracted using the Laemmli buffer. As expected, tricine electrophoresis allowed for a clearer visualization of protein bands below 30 kDa, with a notable emphasis on proteins with a molecular mass below 14 kDa (Figure 3).

### 3.3. Comparative Analysis of Peritrophic Matrix Proteins and Bovine Hemoglobin: Structural and Sequence Insights

**Figure 4:**
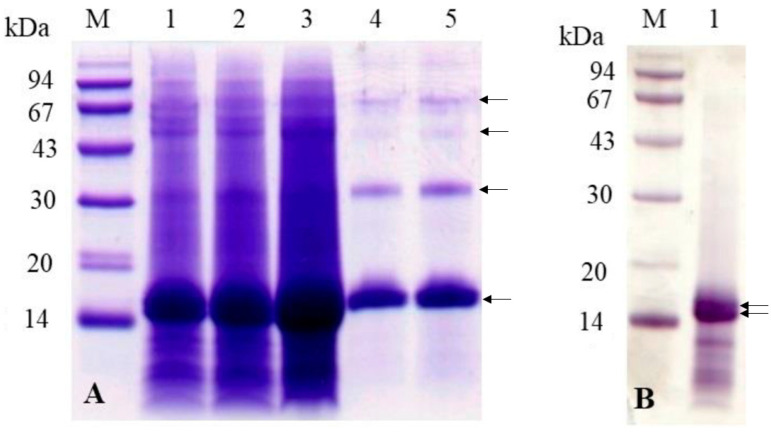
Analysis of *A. aegypti* peritrophic matrix (PM) proteins and commercial bovine hemoglobin by electrophoresis. (**A**) Tricine-SDS-PAGE contained a total concentration of 10% acrylamide and bis-acrylamide (10%T) and a crosslinker ratio of 3% (3%C). The gel was stained with Coomassie blue showing protein profiles from different PM quantities and purified hemoglobin. Lane M: molecular mass markers (94, 67, 43, 30, 20, and 14 kDa); lanes 1–3: proteins extracted from 2, 4, and 8 PMs, respectively; and lanes 4–5: commercial bovine hemoglobin (5 μg and 10 μg). The arrows indicate, from top to bottom, the tetrameric, trimeric, dimeric, and monomeric forms of bovine hemoglobin. (**B**) SDS-PAGE analysis demonstrating the presence of protein dimers in PM extracts. Lane M: molecular mass markers; lane 1: protein extract from a single *A. aegypti* PM. The two arrows indicate the alpha and beta subunits of bovine hemoglobin.

To better understand how proteins from the peritrophic matrix (PM) compare to hemoglobin, we conducted denaturing electrophoresis using a tricine gel. For this analysis, PM proteins were extracted from 2, 4, and 8 PMs of *A. aegypti* mosquitoes (Figure 4A). These mosquitoes were blood-fed and dissected 24 h post-feeding. As controls, we included 5 and 10 µg of bovine hemoglobin (Figure 4A). The results demonstrated that proteins extracted from the PM, with molecular weights of approximately 67, 43, 30, and 14 kDa, exhibited gel migration patterns consistent with the tetrameric form of hemoglobin (67 kDa) and its trimeric (43 kDa), dimeric (30 kDa), and monomeric (14 kDa) forms. Notably, when lower amounts of PM proteins were analyzed (Figure 4A, lane 1) and in Figure 4B, the 14 kDa protein band resolved into two distinct bands with closely similar molecular weights, which are likely the alpha and beta subunits of hemoglobin.

To confirm the identity of the proteins associated with the PM, we analyzed the N-terminal amino acid sequences of the 14 kDa predominant proteins extracted from it. The sequences matched those of the alpha and beta subunits of mouse hemoglobin, consistent with the use of mice as the blood source for the mosquitoes in this experiment. Specifically, the higher molecular weight protein displayed the sequence VLSGEDKSN, corresponding to the alpha subunit, while the lower molecular weight protein showed the sequence VHLTDAEKA, corresponding to the beta subunit.

These sequencing results align with the findings from Western blot analysis, where an anti-hemoglobin antibody strongly recognized the 14 kDa hemoglobin subunits in the PM extracts from blood-fed mosquitoes (Figure S1, Supplementary Materials). Together, these results confirm the association of hemoglobin with the PM and its incorporation into the matrix during blood digestion.

### 3.4. Visualization of Hemoglobin–Chitin Interaction in the Peritrophic Matrix via Immunofluorescence Microscopy

**Figure 5:**
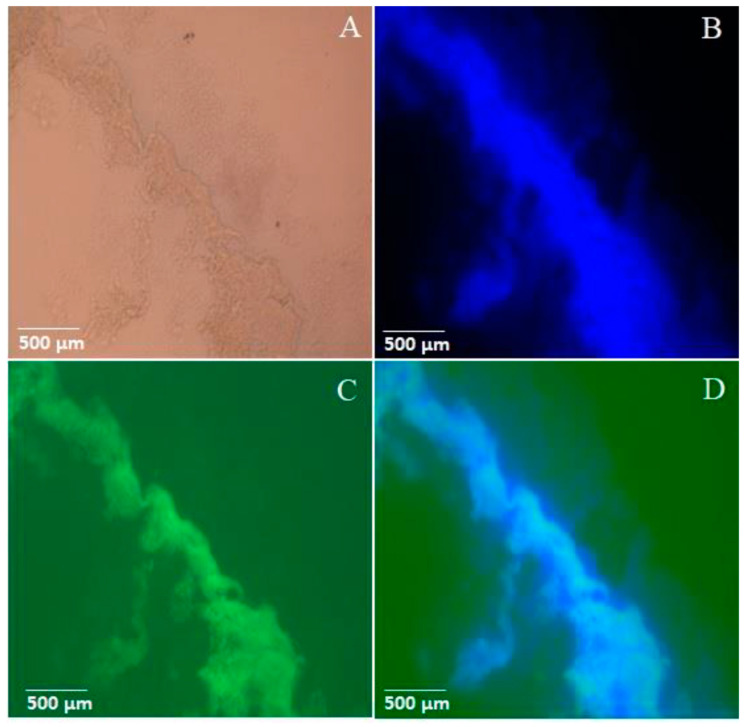
Visualization of hemoglobin–peritrophic matrix interaction in *A. aegypti* after 24 h of artificial feeding (plasma + hemoglobin, 70 mg/mL) using epifluorescence microscopy. (**A**) Differential interference contrast (DIC) showing PM structure; (**B**) PM chitin network visualized by Calcofluor White staining (blue fluorescence); (**C**) immunolocalization of hemoglobin using Alexa Fluor-conjugated secondary antibody (green fluorescence); and (**D**) merged images showing co-localization of chitin and hemoglobin in the PM (ImageJ overlay). All images were captured at the same magnification (scale bar = 500 μm). The fluorescence patterns demonstrate the specific association between hemoglobin and the PM chitin network during blood digestion.

To investigate the interaction between hemoglobin and the peritrophic matrix (PM), the mosquitoes fed with bovine hemoglobin were dissected, and their midguts were subjected to an immunofluorescence analysis. The midguts were treated with a primary anti-human hemoglobin antibody, followed by incubation with a fluorescent secondary antibody that emitted green light. Additionally, the midguts were stained with CalcoFluor White, a chitin-specific dye that fluoresces blue upon binding. Examination under an epifluorescence microscope revealed overlapping fluorescence signals from hemoglobin and chitin along the PM structure (Figure 5). Similar overlapping signals were observed in the PM of blood-fed mosquitoes (Figure S2, Supplementary Materials). In contrast, the PM of mosquitoes fed with albumin showed only CalcoFluor White staining, demonstrating the specificity of the primary anti-human hemoglobin antibody binding (Figure S3, Supplementary Materials).

### 3.5. Affinity Chromatography Profiles of Chitin with Bovine Hemoglobin and Hemin

**Figure 6:**
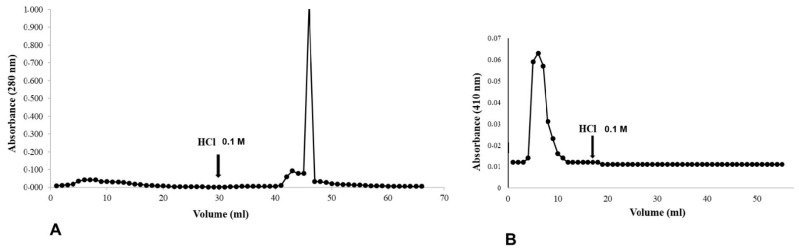
Analysis of chitin-binding properties of hemoglobin and hemin using affinity chromatography. (**A**) Chromatographic profile showing the interaction between hemoglobin and chitin matrix, monitored at 280 nm, demonstrating specific binding as evidenced by the elution peak after addition of 0.1 M HCl. (**B**) Chromatographic profile monitored at 410 nm showing lack of interaction between hemin and chitin matrix, as indicated by the absence of significant elution peak after HCl addition. Both experiments were performed using 0.1 M sodium acetate buffer (pH 6.0) for equilibration. Fractions of 1 mL were collected at one-minute intervals. The results demonstrate that while hemoglobin exhibits specific binding to the chitin matrix, hemin does not show affinity under the same experimental conditions.

The affinity between hemoglobin and heme with chitin was examined by affinitychromatography. The graph (Figure 6A) shows the chromatographic elution profile of bovine hemoglobin. As expected, only one peak was observed, referring to bovine hemoglobin in the eluate with 0.1 M HCl, where the proteins with affinity for chitin are located. In the graph (Figure 6B) referring to the chromatographic elution profile of heme, it can be seen from the presence of a peak in the non-retained fraction that heme shows no affinity for chitin.

### 3.6. Computational Insights into the Binding of Bovine Hemoglobin Subunits with (NAG)_4_ Oligosaccharide: Structural Modeling and Docking Analysis

**Figure 7:**
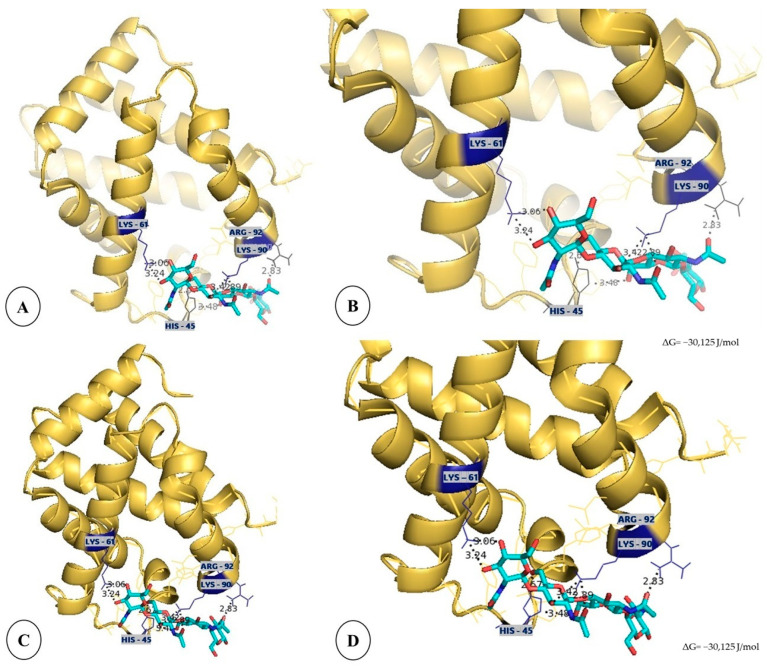
Molecular docking demonstrating the possible molecular interactions between bovine hemoglobin (golden color) and the oligosaccharide (NAG)_4_ (blue-green color) using DockThor v.2 software. (**A**) Three-dimensional structure of the alpha subunit of hemoglobin showing the bond with (NAG)_4_. (**B**) Enlargement of the alpha subunit showing the amino acid residues involved in the interaction: lysine (LYS 61 and LYS 91), arginine (ARG 92) and histidine (HIS 45), with the formation of hydrogen bonds (distances < 4 Å). (**C**) Three-dimensional structure of the beta subunit of hemoglobin showing the interaction with (NAG)_4_. (**D**) Enlargement of the beta subunit highlighting the binding residues, the same ones identified in the alpha subunit, confirming similar interactions with ΔG = −30,125 J/mol.

The molecular docking analysis revealed specific interactions between bovine hemoglobin α and β subunits and the oligosaccharide (NAG)_4_ (Figure 7). Both subunits exhibited an identical binding affinity energy (ΔG) = −30,125 joules/mol (J/mol)., suggesting favorable and spontaneous interactions. The three-dimensional structural models demonstrate that the oligosaccharide binds to specific regions of both hemoglobin subunits through multiple hydrogen bond interactions with key amino acid residues, including lysine (LYS 61 and LYS 90), arginine (ARG 92), and histidine (HIS 45). These residues form stable hydrogen bonds with interatomic distances smaller than 4 Å, indicating strong and stable bonding networks. The binding mode shows a remarkable similarity between α and β subunits, suggesting a conserved interaction mechanism between hemoglobin and chitin oligomers. The structural analysis emphasizes the importance of basic amino acid residues and histidine in establishing specific interactions with the oligosaccharide, providing molecular insights into the mechanism of hemoglobin binding to the chitin-rich peritrophic matrix.

### 3.7. Temporal Dynamics of Hemoglobin Association and Peroxidase Activity in the Peritrophic Matrix of A. aegypti During Blood Digestion

**Figure 8:**
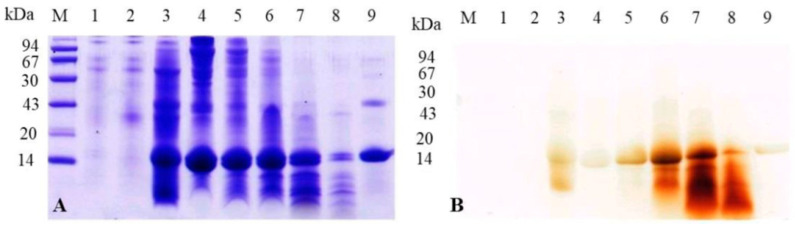
Analysis of hemoglobin binding and peroxidase activity in *A. aegypti* peritrophic matrix during blood digestion. (**A**) Coomassie-stained SDS-PAGE showing protein profiles and (**B**) DMB-stained gel revealing peroxidase activity of PM-associated proteins. Lane M: molecular mass markers (94, 67, 43, 30, 20, and 14 kDa); lanes 1–9: PM samples from mosquitoes fed with: plasma + albumin (1), plasma + hemin (2), plasma + hemoglobin (3), blood samples collected at different time points after feeding—6 h (4), 12 h (5), 24 h (6), 36 h (7), 48 h (8), and commercial bovine hemoglobin (10 μg) as reference (9). Note the distinct 14 kDa hemoglobin band patterns across different feeding conditions and time points, and the corresponding peroxidase activity revealed by DMB staining. The temporal analysis demonstrates the progressive changes in both protein content and enzymatic activity during the digestive process.

An SDS-PAGE analysis was used to assess the dynamics of hemoglobin association with the peritrophic matrix (PM) in *A. aegypti* during blood digestion (Figure 8). The Coomassie-stained gel (Figure 8A) revealed that PM samples from mosquitoes fed with plasma + albumin or plasma + hemin did not exhibit the characteristic hemoglobin subunits (14.4 kDa). In contrast, both plasma + hemoglobin and blood-fed samples showed distinct hemoglobin subunit bands. Blood-fed samples displayed a clear temporal pattern, with strong band intensities observed at 6 h post-feeding, which gradually decreased over subsequent time points, reaching a lower intensity at 48 h (lanes 4–8). The parallel analysis of the peroxidase activity using DMB staining (Figure 8B) revealed distinct temporal patterns. While the PM samples from plasma + albumin and plasma + hemin treatments showed no peroxidase activity, the hemoglobin-containing samples displayed a dynamic profile. The peroxidase activity associated with hemoglobin bands progressively increased from 6 h post-feeding, reaching maximum levels between 24–36 h, followed by a notable decline at 48 h, which coincides with the final stages of blood digestion. The commercial bovine hemoglobin (10 μg) exhibited a relatively low peroxidase activity despite its high protein concentration. Additionally, peroxidase-active bands below 14 kDa, likely representing hemoglobin degradation products, were more prominent at 24 and 36 h (lanes 6 and 7). The inverse relationship between the hemoglobin band intensity and peroxidase activity suggests progressive structural modifications of PM-associated hemoglobin during digestion. While the total protein amount decreases over time, the accessibility of heme groups appears to increase, likely due to partial proteolysis or conformational changes, resulting in enhanced peroxidase activity despite lower protein quantities.

The densitometric analysis of hemoglobin bands associated with the PM revealed a clear temporal pattern during blood digestion (Figure S4A, Supplementary Materials). The analysis showed high band intensities during the early digestion stages (100% at 6 h and 90% at 12 h), with a subsequent decrease to 70% at 24 h. The intensity continued to decline, reaching 57% at 36 h and 22% at 48 h. The densitometric analysis of the peroxidase activity of hemoglobin bands associated with the PM revealed a distinct temporal pattern (Figure S4B, Supplementary Materials). Using blood samples at 24 h as a reference (100%), the analysis showed that the plasma + hemoglobin samples exhibited a relatively low intensity (35%). During blood digestion, the band intensity increased progressively from 55% at 6 h to 80% at 12 h post-feeding, reaching maximum levels at 24 h. Subsequently, the intensity gradually declined to 90% at 36 h and further decreased to 68% at 48 h. The commercial bovine hemoglobin displayed a minimal intensity at approximately 20%. This pattern demonstrates a dynamic profile of peroxidase activity, characterized by a progressive increase during early digestion, followed by a gradual decline during later stages of blood meal processing.

### 3.8. Comparative Analysis of Luminal and Peritrophic Matrix-Associated Hemoglobin Degradation During Blood Digestion in A. aegypti

**Figure 9:**
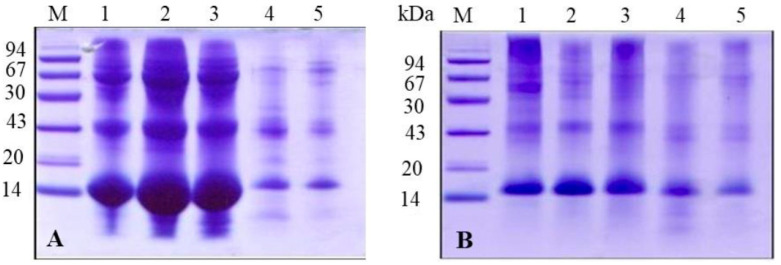
Comparative analysis of hemoglobin degradation in *A. aegypti* luminal content and peritrophic matrix during blood digestion by SDS-PAGE (15%). (**A**) Protein profile of luminal hemoglobin and (**B**) PM-associated hemoglobin showing temporal changes in protein content. Lane M: molecular mass markers (94, 67, 43, 30, 20, and 14.4 kDa); lanes 1–5: samples collected at different time points after blood feeding—6 h (1), 12 h (2), 24 h (3), 36 h (4), and 48 h (5). Note the differential degradation patterns between luminal and PM-associated hemoglobin, particularly the 14.4 kDa band intensity changes over time.

The comparative analysis of luminal and peritrophic matrix (PM)-associated hemoglobin (14 kDa) degradation during blood digestion in *A. aegypti* reveals distinct patterns of protein breakdown (Figure 9). The SDS-PAGE analysis shows that luminal hemoglobin (Figure 9A) exhibits more intense 14 kDa bands during the first 24 h (lanes 1–3), followed by a sharp decline in intensity at 36 and 48 h (lanes 4–5). In contrast, PM-associated hemoglobin (Figure 9B) shows a more gradual decrease in band intensity, with moderate intensity levels maintained even at later time points.

The densitometric analysis corroborates these observations, highlighting that luminal hemoglobin undergoes faster degradation compared to PM-associated hemoglobin (Figure S5). Specifically, luminal hemoglobin exhibits a pronounced reduction in band intensity over time: from 100% at 6 h (reference) to approximately 92% at 12 h, 87% at 24 h, a sharp drop to 45% at 36 h, and a further decrease to 32% at 48 h. This suggests that luminal hemoglobin is more rapidly degraded during blood digestion (Figure S5A). In contrast, the PM-associated hemoglobin shows a more gradual decrease in intensity: it starts at 100% at 6 h (reference), decreases slightly to 97% at 12 h and around 95% at 24 h, and declines mildly to 92% at 36 h, with a moderate reduction to 88% at 48 h (Figure S5B).

These findings indicate that luminal hemoglobin is subject to faster degradation, with a sharp decline after 24 h, while PM-associated hemoglobin remains relatively stable throughout digestion. This suggests that the association with the peritrophic matrix provides some level of protection against proteolysis, contributing to a slower degradation rate for hemoglobin within the matrix during blood digestion.

### 3.9. Proteolytic Regulation of Heme Protein Association with A. aegypti Peritrophic Matrix: A Comparative SDS-PAGE Analysis

**Figure 10:**
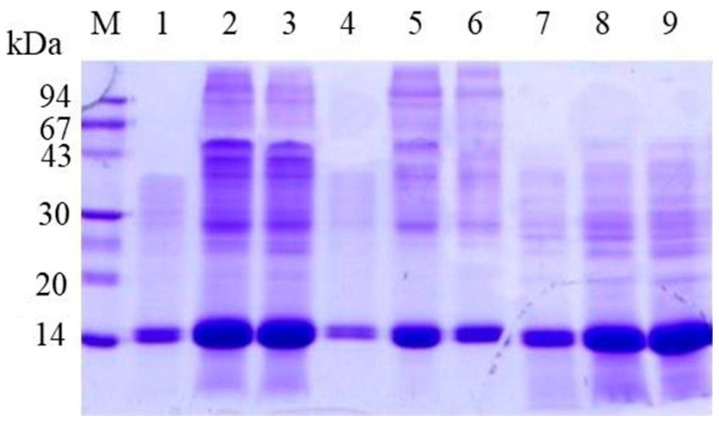
Temporal analysis of proteolytic effects on hemoglobin binding to Aedes aegypti peritrophic matrix. Proteins were separated by 15% SDS-PAGE and stained with Coomassie brilliant blue. Lane M: molecular mass standards (94, 67, 43, 30, 20, and 14 kDa). Time-course analysis of peritrophic matrix protein profiles from mosquitoes fed with blood alone (lanes 1–3; 2 h, 4 h, and 6 h post-feeding), blood supplemented with exogenous trypsin (lanes 4–6; 2 h, 4 h, and 6 h post-feeding), or blood containing trypsin inhibitor (lanes 7–9; 2 h, 4 h, and 6 h post-feeding). The electrophoretic profile demonstrates differential hemoglobin association with the peritrophic matrix under varying proteolytic conditions, with prominent 14 kDa bands showing enhanced preservation in trypsin-inhibited samples and reduced intensity in trypsin-treated samples compared to controls.

The SDS-PAGE analysis reveals distinct patterns in the 14 kDa hemoglobin band across different experimental treatments, providing insights into the dynamics of hemoglobin–peritrophic matrix interactions in *A. aegypti* (Figure 10). In the control blood-fed mosquitoes (lanes 1–3), the 14 kDa band exhibits a clear temporal progression, with the band intensity increasing substantially from 2 to 6 h post-feeding. This progressive accumulation suggests a time-dependent incorporation of hemoglobin into the peritrophic matrix during the early phases of blood digestion. When examining the effect of proteolytic activity, the samples treated with exogenous trypsin (lanes 4–6) display a markedly reduced intensity of the 14 kDa band compared to the controls. This reduction is particularly evident across all time points, indicating that enhanced proteolytic activity significantly impairs hemoglobin association with the peritrophic matrix. The diminished band intensity suggests that trypsin actively degrades hemoglobin before it can establish stable interactions with the matrix. Conversely, when the proteolytic activity was suppressed using trypsin inhibitor (lanes 7–9), the 14 kDa band maintained a robust intensity throughout the experimental time course. The preservation of a strong band intensity under these conditions demonstrates that the inhibition of proteolytic activity effectively protects hemoglobin from degradation, allowing for sustained association with the peritrophic matrix. These findings collectively demonstrate that the association of hemoglobin with the peritrophic matrix is dynamically regulated by proteolytic activity, with proteolysis serving as a key modulator of this interaction.

The bar graph (Figure S6) quantifies the intensity of the 14 kDa hemoglobin band across treatments, using plasma + hemoglobin as the reference (100%). The control blood samples show high intensities at early time points (92% at 2 h, 90% at 4 h, and 85% at 6 h). In contrast, the trypsin-treated samples exhibit significantly lower intensities (25% at 2 h, 20% at 4 h, and 18% at 6 h). Notably, the trypsin inhibitor-treated samples maintain high band intensities throughout the experiment (80% at 2 h, 95% at 4 h, and 100% at 6 h). This quantitative analysis confirms that the trypsin inhibitor effectively preserves the hemoglobin association with the peritrophic matrix, providing protection against enzymatic degradation.

### 3.10. Analysis of Heme Protein Binding to Aedes aegypti Peritrophic Matrix by SDS-PAGE

**Figure 11:**
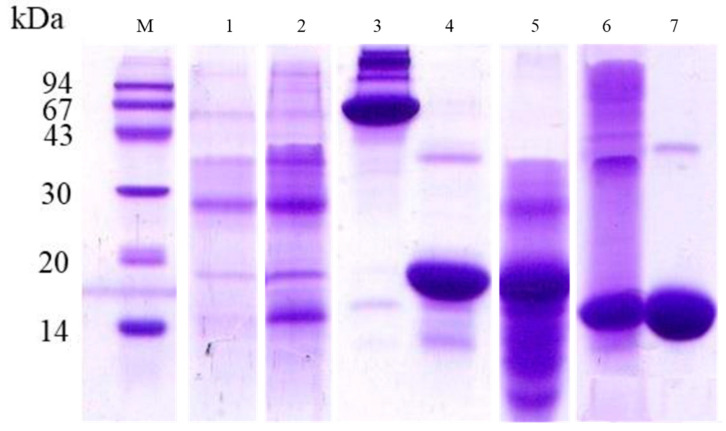
Protein binding analysis of heme and non-heme proteins to *A. aegypti* peritrophic matrix by SDS-PAGE. Proteins were separated on 15% polyacrylamide gel and stained with Coomassie brilliant blue. Lane M: molecular mass standards (94, 67, 43, 30, 20, and 14 kDa). Peritrophic matrix protein profiles from mosquitoes fed with plasma alone (lane 1), plasma supplemented with albumin (lane 2), purified commercial albumin (lane 3), commercial myoglobin (lane 4), plasma supplemented with myoglobin (lane 5), plasma supplemented with cytochrome C (lane 6), and commercial cytochrome C (lane 7). The electrophoretic profile demonstrates selective binding of heme proteins (myoglobin and cytochrome C) to the peritrophic matrix, while non-heme proteins like albumin show no specific association.

The 15% SDS-PAGE analysis stained with Coomassie blue reveals interesting interaction patterns between proteins and the peritrophic matrix (PM) of *A. aegypti* (Figure 11) The gel displays a molecular weight marker (lane M) with proteins ranging from 14 to 94 kDa as reference standards. In lanes 1 and 2, where the PM samples from mosquitoes fed with plasma and plasma plus albumin were applied, respectively, very similar protein profiles with faint bands are observed, suggesting that albumin was degraded without associating with the PM. As a control, lane 3 shows the commercial albumin with an intense band at approximately 67 kDa. The commercial myoglobin (lane 4) exhibits a prominent band around 20 kDa, and this same pattern is observed in lane 5, where the PM samples from mosquitoes fed with plasma plus myoglobin were applied, indicating specific association of this protein with the PM. Similarly, the commercial cytochrome C (lane 7) shows a characteristic band at approximately 14 kDa, which is also observed in lane 6, corresponding to the PM from mosquitoes fed with plasma plus cytochrome C. These results clearly demonstrate that heme-containing proteins, such as myoglobin and cytochrome C, are capable of specifically associating with the PM, unlike albumin, which does not exhibit this capability.

## 4. Discussion

The PM is a multifaceted structure critical to mosquito blood digestion, serving as both a physical barrier and a biochemical regulator. Its antioxidant role is particularly significant, as the PM binds a large proportion of free heme, thereby protecting the intestinal epithelium from oxidative damage. PM synthesis begins 2–6 h post-blood ingestion and is fully formed by 24 h, persisting for approximately 48 h before being excreted [23,33].

Our findings highlight the dynamic interaction between hemoglobin and the PM during blood digestion. Confocal microscopy revealed the specific and organized binding of hemoglobin to chitin within the PM, with hemoglobin localized throughout the PM structure. This binding appears to occur via strong and stable hydrogen bonds, as confirmed by the molecular docking analysis. The interactions involve key residues in the alpha and beta subunits of hemoglobin, forming bonds with chitin oligosaccharides (NAG)_4_ at distances less than 4 Å, which are essential for maintaining the stability of the hemoglobin–chitin complex [34,35].

The electrophoretic analysis of PM proteins revealed several prominent bands, particularly at approximately 43, 30, and 14 kDa, which corresponded to hemoglobin subunits. Additionally, the tetrameric form of hemoglobin was also observed in the gels, albeit as a weaker band, suggesting its lower abundance compared to the subunits. Interestingly, the 14 kDa band resolved into two distinct proteins, corroborating the tetrameric structure of human hemoglobin composed of two alpha and two beta subunits, each with a molecular weight of around 17 kDa [36,37]. These results suggest that hemoglobin association with the PM may provide structural stability similar to that conferred by peritrophins, key PM proteins that interact with chitin via chitin-binding domains [17,38].

The temporal regulation of the hemoglobin–PM interaction further underscores the complexity of this relationship. Hemoglobin preferentially binds to the PM during the first hours post-blood feeding, a period characterized by low proteolytic activity. This early interaction coincides with PM formation, ensuring that hemoglobin associated with the PM is protected from luminal proteolysis during the later stages of digestion. Additionally, literature reports showing the presence of proteolytic enzymes associated with the PM of mosquitoes [39,40] indicate the potential for localized hemoglobin hydrolysis directly at the PM surface. This spatial organization would establish an efficient system where the released heme could be immediately sequestered by binding sites within the PM, thereby reducing the free heme concentration in the midgut lumen and minimizing oxidative stress. As hemoglobin is hydrolyzed in the PM, additional hemoglobin could be dynamically recruited to the matrix in a controlled process of heme release and sequestration, creating a sustained mechanism for heme detoxification.

Affinity chromatography and molecular docking studies confirmed that hemoglobin binds directly to chitin, while hemin alone lacks affinity for the PM, suggesting that the protein moiety drives this interaction. Interestingly, other heme-containing proteins, such as myoglobin and cytochrome C, also exhibited binding to the PM, likely mediated by peritrophins with heme-binding domains [22]. These findings support a dual mechanism of interaction: direct protein–chitin binding and mediation through heme-binding peritrophins. The peroxidase-like activity of hemoglobin further highlights the functional implications of their association with the PM. By stabilizing these proteins within its structure, the PM not only regulates heme release but also mitigates oxidative stress, maintaining gut homeostasis during digestion.

Another notable observation was the greenish coloration of the midgut 72 h post-feeding, indicative of biliverdin synthesis catalyzed by heme oxygenase, which breaks down heme into biliverdin, Fe, and carbon monoxide [41,42]. This suggests that at later stages of digestion, when the PM begins to degrade, residual heme is processed by the intestinal epithelium.

These findings illustrate the intricate interplay between hemoglobin and the PM, with implications for mosquito physiology and vector control. The ability of hemoglobin to contribute to PM stability and function underscores its importance in mosquito digestion, while the selective binding mechanisms provide potential targets for disrupting mosquito survival. Further research should explore these molecular interactions to identify innovative strategies for mosquito control.

## 5. Conclusions

This study offers valuable insights into the structural and functional roles of the PM in *A. aegypti*. Far from being a simple passive barrier, the PM emerges as an active player in the digestion process, forming specific and organized interactions with hemoglobin. Through confocal microscopy, molecular docking, and biochemical analyses, we demonstrated that hemoglobin binds to the PM in a highly structured manner, driven by direct protein–chitin interactions and mechanisms mediated by peritrophins.

The differences in degradation patterns between luminal and PM-associated hemoglobin highlight the protective role of the PM, by protecting hemoglobin from luminal proteolysis while allowing for its localized and more coordinated degradation at the PM, thereby regulating the release of heme. The PM not only shields the gut from oxidative damage, but also maintains its structural integrity. This dual role underscores the PM’s importance as a critical adaptation for blood-feeding mosquitoes.

Additionally, this study sheds light on the specificity of protein–chitin interactions within the PM. Hemoglobin’s strong and selective binding to the PM points to a mechanism that could be leveraged for mosquito control. Disrupting these interactions or impairing PM formation could interfere with mosquito digestion and reduce their survival, offering promising avenues for combating mosquito-borne diseases.

Overall, our findings deepen the understanding of mosquito physiology and open new possibilities for innovative vector control strategies, paving the way for improved disease management and prevention efforts.

## Data Availability

The original contributions of this study are detailed in the article. For additional information or inquiries, please contact the corresponding authors.

## Supplementary Materials

The following supporting information can be downloaded at https://www.mdpi.com/article/10.3390/insects16020116/s1, Figure S1. Western blotting of proteins extracted from adult *A. aegypti* PM and revealed with an antibody against human hemoglobin. Lane 1, proteins extracted from PM of blood-fed mosquitoes; lane 2, commercial bovine hemoglobin (10 µg). The arrow indicates Hb monomers. Figure S2. Z-stack confocal microscopy analysis showing the spatial distribution of hemoglobin and chitin in *A. aegypti* peritrophic matrix (PM) after 24 h of blood feeding with sheep blood. Sequential optical sections (0.3 μm intervals) demonstrate the three-dimensional organization of the PM structure. Blue fluorescence indicates chitin network labeled with Calcofluor White, and green fluorescence shows immunolocalized hemoglobin detected using Alexa Fluor-conjugated goat anti-rabbit IgG. Images were acquired using ZEISS confocal microscope and processed with ZEISS Zen Lite software. Scale bars = 5 μm. The sequential z-stack analysis provides a comprehensive three-dimensional visualization of the peritrophic matrix architecture, demonstrating how hemoglobin molecules integrate within the chitin network. By examining consecutive optical sections, we can observe the intricate spatial distribution of both components throughout different PM layers. Figure S3. Visualization of peritrophic matrix (PM) in *A. aegypti* midgut 24 h after artificial feeding (plasma + albumin, 70 mg/mL) using epifluorescence microscopy. (A) Differential interference contrast (DIC) showing PM structure; (B) PM chitin network visualized by Calcofluor White staining (blue fluorescence); and (C) no specific fluorescence detected using anti-human hemoglobin antibody and Alexa Fluor-conjugated secondary antibody, demonstrating absence of hemoglobin binding to the PM under these feeding conditions. All images were captured at the same magnification (scale bar = 500 μm). The lack of green fluorescence in panel C indicates that hemoglobin does not associate with the PM chitin network when mosquitoes are fed an artificial meal lacking hemoglobin. Figure S4. Densitometric analysis of hemoglobin bands associated with *A. aegypti* peritrophic matrix. (A) Band intensity analysis of Coomassie-stained gel and (B) DMB-stained gel showing peroxidase activity. Values are expressed as percentage relative to plasma + hemoglobin (100%). Samples include plasma + hemoglobin (reference), blood samples collected at different time points (6 h, 12 h, 24 h, 36 h, and 48 h after feeding), and commercial bovine hemoglobin. The graphs show a progressive decrease in both protein content and peroxidase activity throughout the digestive process. Figure S5. Densitometric analysis of luminal and PM-associated hemoglobin during blood digestion in *A. aegypti.* (A) Band intensity of luminal hemoglobin showing rapid degradation after 24 h, with values dropping from 100% (6 h) to approximately 32% (48 h). (B) Band intensity of PM-associated hemoglobin demonstrating gradual decrease over time, maintaining approximately 88% of initial intensity at 48h. Values are expressed as percentage relative to 6h samples (100%). Measurements were obtained from SDS-PAGE analysis of samples collected at 6 h, 12 h, 24 h, 36 h, and 48 h after blood feeding. Figue S6. Densitometric analysis of hemoglobin (14 kDa) associated with *A. aegypti* peritrophic matrix under different treatments. Band intensities are expressed as percentage relative to trypsin inhibitor 6 h (100%). Samples include blood-fed controls (2 h, 4 h, and 6 h), trypsin-treated samples (2 h, 4 h, and 6 h), and trypsin inhibitor-treated samples (2 h, 4 h, and 6 h). The graph demonstrates reduced hemoglobin association in trypsin-treated samples and enhanced association in trypsin inhibitor-treated samples compared to blood-fed controls.
