# Supplementary material for: Structural and Functional Analysis of Hemoglobin Binding to the Peritrophic Matrix During Blood Digestion in Aedes aegypti
Source: Insects. 2025 Jan 24;16(2):116. doi: 10.3390/insects16020116 (PMC11855609; doi:10.3390/insects16020116)
Supplement: Supplementary file 1 [file insects-16-00116-s001.zip › insects-3388418-supplementary.pdf]

Supplementary material

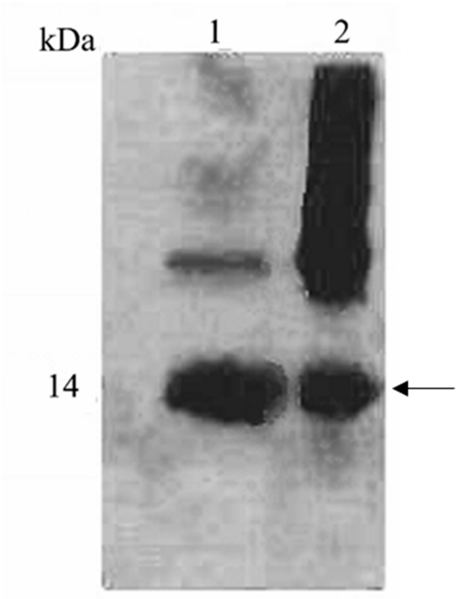

Figure S1. Western blotting of proteins extracted from adult *A. aegypti* PM and revealed with an antibody against human hemoglobin. Lane 1, proteins extracted from PM of blood-fed mosquitoes; lane 2, commercial bovine hemoglobin (10 μg). The arrow indicates Hb monomers.

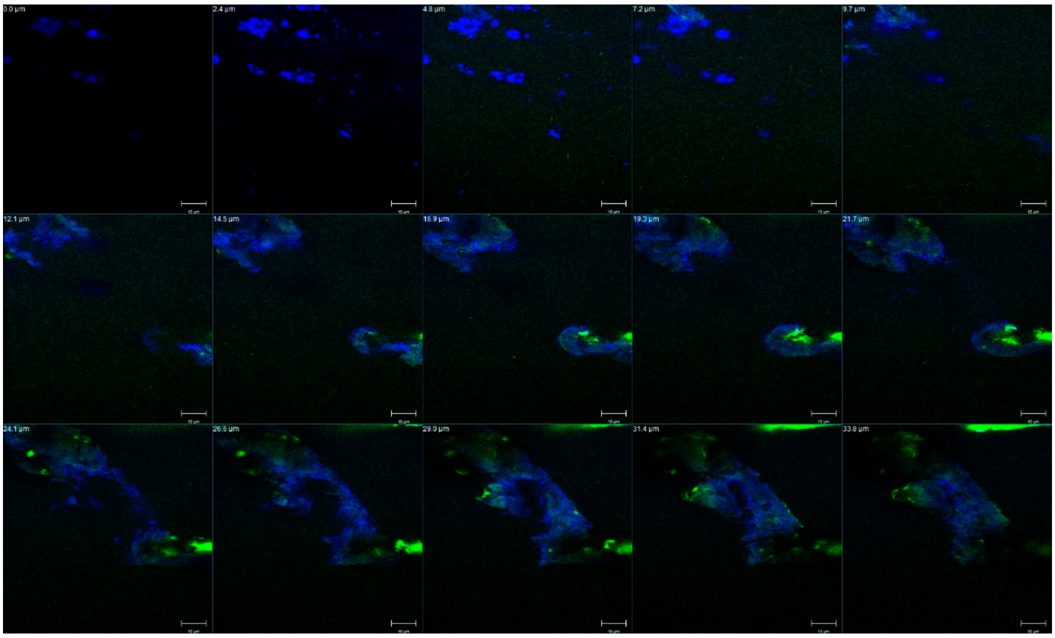

Figure S2. Z-stack confocal microscopy analysis showing the spatial distribution of hemoglobin and chitin in *A. aegypti* peritrophic matrix (PM) after 24 hours of blood feeding with sheep blood. Sequential optical sections (0.3 µm intervals) demonstrate the three-dimensional organization of the PM structure. Blue fluorescence indicates chitin network labeled with Calcofluor White, and green fluorescence shows immunolocalized hemoglobin detected using Alexa Fluor-conjugated goat anti-rabbit IgG. Images were acquired using ZEISS confocal microscope and processed with ZEISS Zen Lite software. Scale bars = 5 µm. The sequential z-stack analysis provides a comprehensive three-dimensional visualization of the peritrophic matrix architecture, demonstrating how hemoglobin molecules integrate within the chitin network. By examining consecutive optical sections, we can observe the intricate spatial distribution of both components throughout different PM layers.

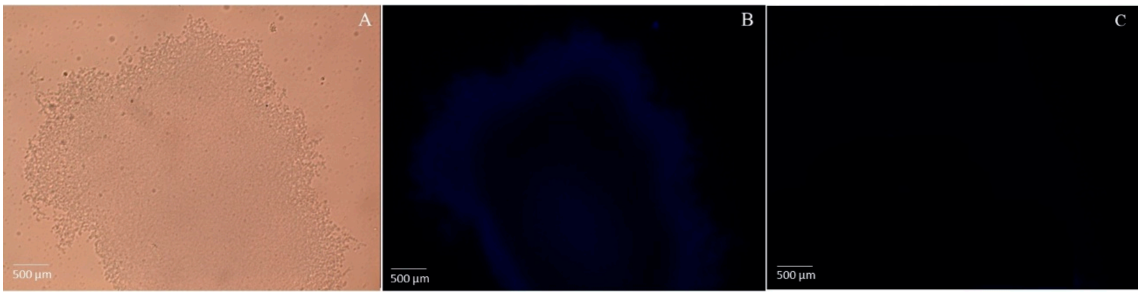

Figure S3. Visualization of peritrophic matrix (PM) in *A. aegypti* midgut 24 hours after artificial feeding (plasma + albumin, 70 mg/mL) using epifluorescence microscopy. (A) Differential interference contrast (DIC) showing PM structure; (B) PM chitin network visualized by Calcofluor White staining (blue fluorescence); (C) No specific fluorescence detected using anti-human hemoglobin antibody and Alexa Fluor-conjugated secondary antibody, demonstrating absence of hemoglobin binding to the PM under these feeding conditions. All images were captured at the same magnification (scale bar = 500 µm). The lack of green fluorescence in panel C indicates that hemoglobin does not associate with the PM chitin network when mosquitoes are fed an artificial meal lacking hemoglobin.

A

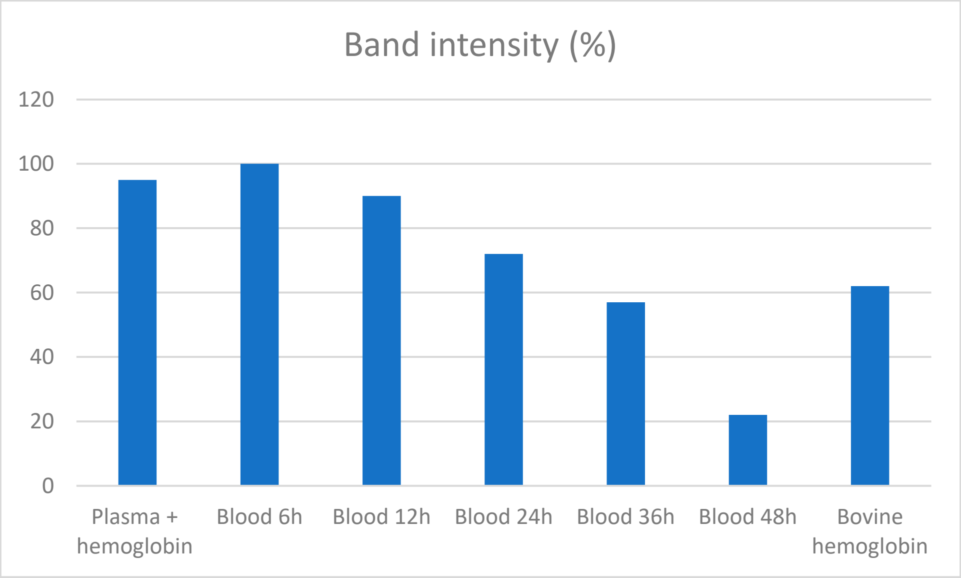

B

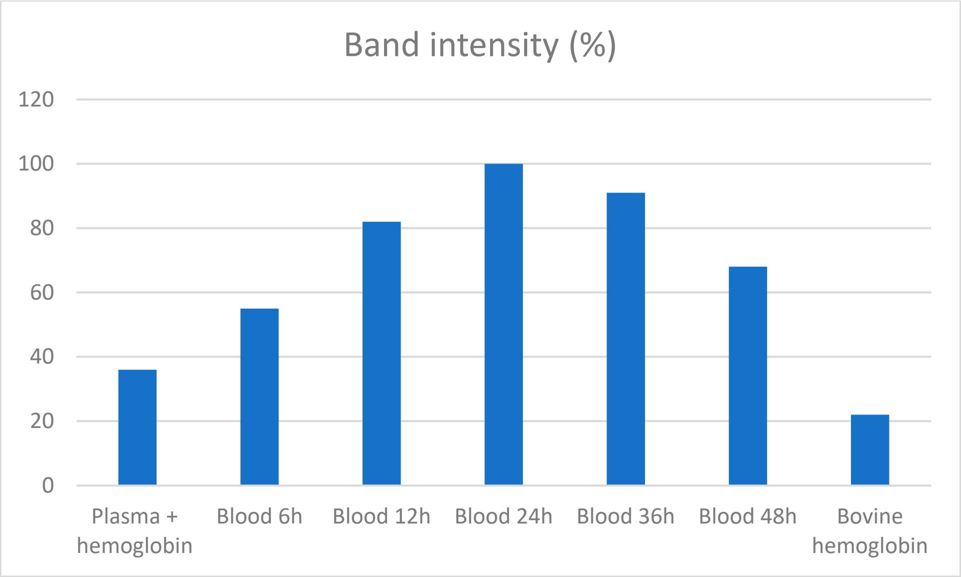

Figure S4. Densitometric analysis of hemoglobin bands associated with *A. aegypti* peritrophic matrix. (A) Band intensity analysis of Coomassie-stained gel and (B) DMB-

stained gel showing peroxidase activity. Values are expressed as percentage relative to plasma + hemoglobin (100%). Samples include plasma + hemoglobin (reference), blood samples collected at different time points (6h, 12h, 24h, 36h, and 48h after feeding), and commercial bovine hemoglobin. The graphs show a progressive decrease in both protein content and peroxidase activity throughout the digestive process.

A

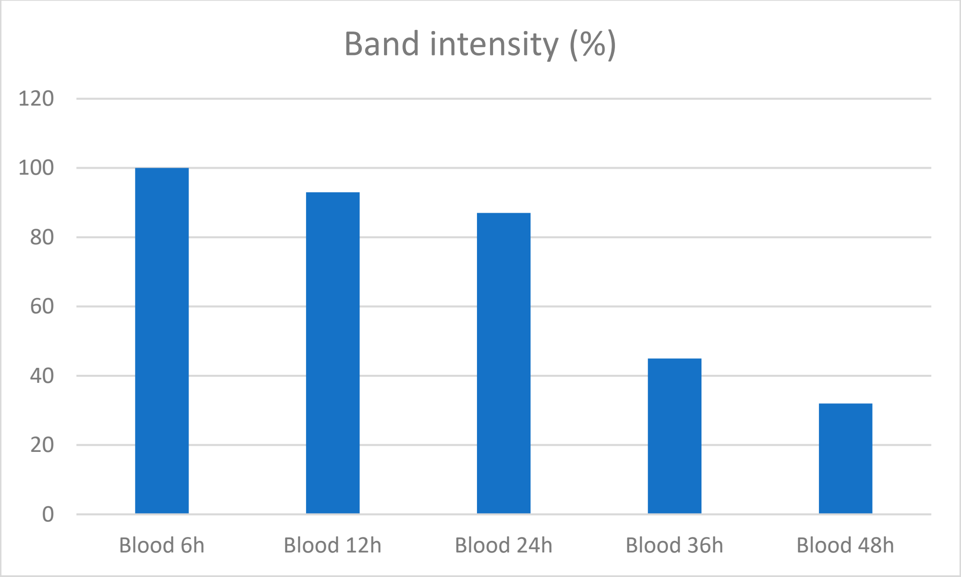

B

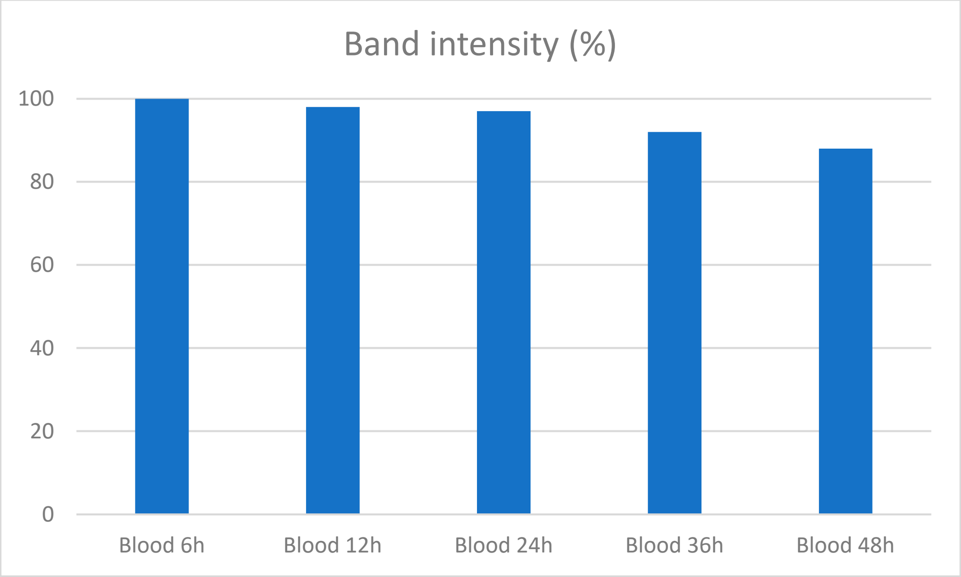

Figure S5. Densitometric analysis of luminal and PM-associated hemoglobin during blood digestion in *A. aegypti.* (A) Band intensity of luminal hemoglobin showing rapid degradation after 24h, with values dropping from 100% (6h) to approximately 32% (48h). (B) Band intensity of PM-associated hemoglobin demonstrating gradual decrease over time, maintaining approximately 88% of initial intensity at 48h. Values are expressed as percentage relative to 6h samples (100%). Measurements were obtained from SDS-PAGE analysis of samples collected at 6h, 12h, 24h, 36h, and 48h after blood feeding.

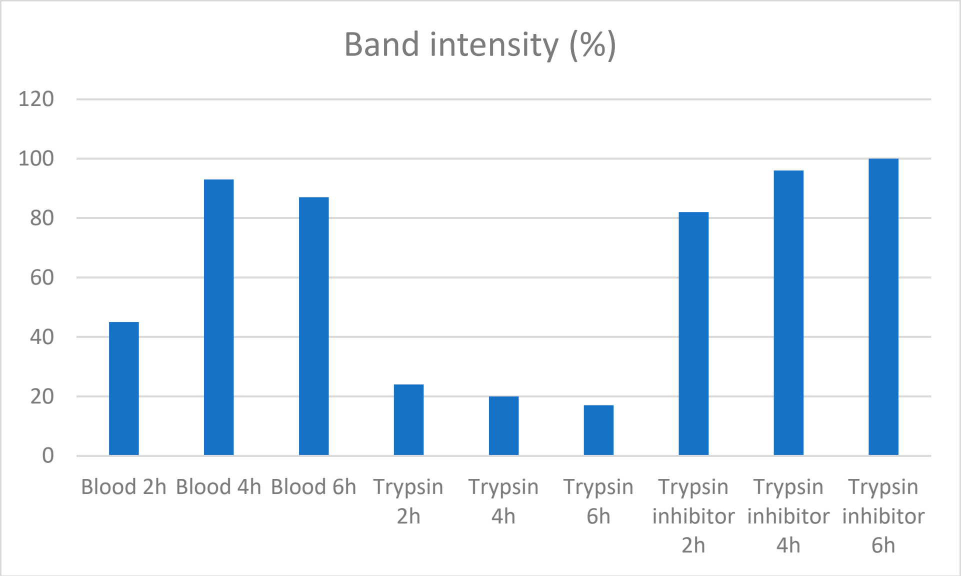

Figue S6. Densitometric analysis of hemoglobin (14 kDa) associated with *A. aegypti* peritrophic matrix under different treatments. Band intensities are expressed as percentage relative to trypsin inhibitor 6h (100%). Samples include blood-fed controls (2h, 4h, and 6h), trypsin-treated samples (2h, 4h, and 6h), and trypsin inhibitor-treated samples (2h, 4h, and 6h). The graph demonstrates reduced hemoglobin association in trypsin-treated samples and enhanced association in trypsin inhibitor-treated samples compared to blood-fed controls
